# Antenatal care follow-up decreases the likelihood of cultural malpractice during childbirth and postpartum among women who gave birth in the last one-year in Gozamen district, Ethiopia: a community-based cross-sectional study

**DOI:** 10.1186/s13690-022-00814-5

**Published:** 2022-02-15

**Authors:** Yeshinat Lakew Ambaw, Birhanu Wubale Yirdaw, Mulunesh Abuhay Biwota, Abebayehu Melesew Mekuryaw, Birhan Tsegaw Taye

**Affiliations:** 1 School of Nursing and Midwifery, Asrat Woldeyes Health Sciences Campus, Debre Berhan University, Debre Berhan, Ethiopia; 2 Department of Clinical Midwifery, School of Midwifery, College of Medicine and Health Sciences, University of Gondar, Gondar, Ethiopia; 3 Department of Women’s and Family Health, School of Midwifery, College of Medicine and Health Sciences, University of Gondar, Gondar, Ethiopia

**Keywords:** Antenatal care, Cultural malpractice, Childbirth and postpartum, Ethiopia

## Abstract

**Background:**

Recent evidence has found widespread reports of women experiencing cultural malpractice during childbirth in Asia and sub-Saharan African countries. Despite an endeavor made to drop maternal and neonatal mortality, Ethiopia remains amongst the highest mortality rate. Thus, strengthening antenatal care (ANC) follow-up is the midst of cultural malpractice reduction during childbirth. This study was aimed to assess the magnitude of cultural malpractice and associated factors during childbirth and postpartum among women who gave birth within one year in Gozamen district, Ethiopia.

**Methods:**

A community-based cross-sectional study was conducted from November 1st to December 30th, 2019. A two-stage sampling technique was used to get a total of 600 women who gave birth within the last one year. Data were collected by using a semi-structured and pretested questionnaire. Then, data were entered into Epi info version 7.0 and exported to SPSS version 25 for analysis. Both bivariate analysis and a multivariable logistic regression model were fitted. The level of significance was declared based on the adjusted odds ratio (AOR) with its 95% confidence interval (CI) and a p-value of ≤0.05.

**Result:**

The Overall magnitude of cultural malpractices during childbirth and postpartum was found to be 31.2% (95%CI: 27.8, 34.7). Out of 600 women, 203(33.9%) were delivered at home, 67(11.2%) practiced abdominal massage, 31(16.6%) avoided colostrum, 24(12.8%) practiced pre-lacteal feeding and 138 (23%) washed their baby before 24 h after delivery. Mothers who have antenatal follow-up (AOR=0.52 95%CI 0.28, 0.94), married marital relation (AOR=0.24, 95%CI:0.07, 0.89), being farmer husband occupation (AOR=6.25 (95%CI: 1.22, 30.30), parity ≥5 (AOR=5, 95%CI: 2.44, 9.52), had significant association with cultural malpractice during childbirth and postpartum.

**Conclusions:**

This study showed there is an improvement in the magnitude of cultural malpractices during childbirth and postpartum, but still it’s high as compared to the country’s maternal health service utilization aim. A well-enforced health education program by well-trained healthcare personnel besides sufficient number of ANC visits are needed to overcome these cultural practices. Further, intervention modalities health education communication outreach programs would be very important to reduce the prevalence of cultural malpractices in the community.

## Background

In human history, the general public has had particular perceptions of health and disease rooted in their own culture which have led to a plurality of practices for disease prevention and cure especially during childbirth and postpartum period [1, 2]. Cultural malpractices reflect values and beliefs held by members of a community for periods, often spanning generations that harm all individuals [3, 4]. During childbirth, it is still a major health problem worldwide specifically in developing countries including Ethiopia that contributes 5-15% of maternal deaths [5]. Women all over the world are facing up too many difficult choices during childbirth. Moreover, cultural practices, beliefs, and taboos are often implicated in determining the care received by mothers during childbirth which is an important determinant of maternal and child mortality [6–8].

Investing in ANC is often positioned as a cost-effective intervention for enhancing maternal and child health, improving the health-seeking behavior of women, and contributing to sustainable development globally. Thus, world health organization (WHO) recommends ANC for a positive pregnancy experience and strength of the relationship(s) between ANC use, and pregnancy outcomes, women’s socioeconomic status, and sustainable development [9]. It aids pregnant women and their families to become arranged and prepared for the consequences related to pregnancy like psychological, social, and financial problems [10]. So, the primary purpose of ANC is to ascertain pregnant women who are at risk, support them to continue healthy, and prevent disease and harmful cultural practices that improve healthcare access throughout the continuity of care [11, 12] Evidence support that ANC enhances women’s knowledge on maternal and neonatal danger signs, [13, 14] boost proper health-seeking practice during perinatal periods [15].

Despite global efforts to combat maternal mortality, 99% of the maternal mortality occurs in developing countries, with sub-Saharan African countries bookkeeping for about 66% of the annual global burden [16–18]. The large magnitude of maternal death is associated and cultural malpractice as a consequence of low utilization [19]. This significant maternal mortality and morbidity can be reduced by strengthening ANC follow-up.

Cultural malpractice during childbirth, low social status, gender discrimination, and lack of awareness in ANC follow-up nearby health facilities are direct and indirect causes of maternal mortality [20]. Harmful cultural practices that affect children and women are female genital mutilation, food taboo, uterine massage (Kneading and squeezing a woman’s abdomen to induce labor), keeping babies from exposure to the sun, newborn bath soon after birth, and feeding newborns babies with fresh butter [6, 21–24]. Despite their harmful nature and their violation of international human rights and laws, cultural malpractice during childbirth persist because they are not questioned and take on an aura of morality in the eyes of those practicing them. As early as the 1950 s, United Nations (UN) specialized agencies and human rights bodies began considering the question of harmful traditional practices affecting the health of women [25]. But these issues have not received consistent broader consideration, and action to bring about any substantial change has been slow [25, 26]. UN agencies and human rights bodies started addressing harmful traditional practices in the early 1990 s but there was little progress.

Some studies tried to show the association between cultural malpractices and place of delivery; women who gave birth at home 28.44% washed their babies immediately after birth. Besides, 88% practiced colostrum avoidance at home versus 4.86% health facility deliveries in northwest Ethiopia [27]. Likewise, 15.9% and 5.9% of women avoided colostrum at home deliveries and institutional deliveries respectively in Kobo district [28].

Nowadays some important international instruments are endorsed by most governments and could serve as a basis for a struggle against cultural malpractices. However, in Ethiopia, there is a significant burden of maternal and neonatal death due to low healthcare utilization. Thus, the national prevalence of home delivery in Ethiopia was 73% with significant variations across regions ranging from 3% in Addis Ababa to 85% in Afar and follow the cultural way of life during childbirth [29–31]. Low healthcare service utilization and cultural way of life generate problems associated with aspects of traditional believes at each individual, community, and household level during delivery and postpartum period [7, 32]. As a result, different cultural practices have been under taken during child birth and postpartum periods in almost all the regions of Ethiopia. There is still a gap which clearly document the types and prevalence of cultural malpractices and influencing factors and put base line for further research in order to recommend the necessary intervention to concerned bodies. Therefore, the main purpose of this research was to assess the prevalence and factors associated with cultural malpractices during childbirth and postnatal period (i.e. abdominal massage, home delivery, colostrum avoidance, pre-lacteal feeding, and bathing newborn soon after birth) in Gozamen district, Ethiopia.

.

## Methods

### Study design, period, and setting

A community-based cross-sectional study was conducted among 600 mothers, who gave birth within the last one year from November 1st to December 30th, 2019 in Gozamen district, Ethiopia. Gozamen district is one of the 18 districts in East Gojjam Zone and the relative location is 300 km away from the capital city of Ethiopia and 260 km from the Regional capital city, Bahir Dar. The district is divided into 31 Kebeles of which 7 are urban and 24 rural Kebeles. The population size of the Woreda is estimated to be 151,312. Among the total population, about 35,679 are women of the reproductive age group. The district has six health centers and 26 health posts.

### Sample size determination

The sample size was determined by using single population proportions based on the following assumptions. Significance level calculated at 95% Confidence level, Margin of error (w) = 5%, Prevalence (P) cultural malpractice during delivery was 37.9% from a previous study.

$$n=\frac{Z\alpha /2{)}^{2}p\left(\text{q}\right)}{{\left(w\right)}^{2}}=\frac{\left(1.96{)}^{2}\text{*}0.379\right(0.621)}{\left(0.05\right)2}= 361.66$$

We added a 10% expected non-response rate which became 398 and a design effect of 1.5, the final sample size found to be 597.

### Sampling technique and procedures

A two-stage sampling technique was applied to get study participants. The Kebeles of Gozamen district were 24 which contain 5378 eligible households. In the first stage, we have used the simple random sampling technique to select five Kebeles from the site (i.e. Assabo, Leklekita, Yetjan, Woynma, Chertekel). In the second stage, we used a cluster sampling technique to select the study households with women who gave birth in the last twelve months. A house-to-house visit was carried out in the selected clusters to find eligible women for the study. All eligible women in the selected clusters were interviewed. If a woman was absent from the household for two consecutive visits considered as a non-response and went the next neighbor household. All the participants who participated were informed verbally about the study and those who gave anonymous written informed consent were enrolled. Finally, due to the cluster effect, the sample size was raised by 20 participants from the predetermined sample. Of 617 asked participants, 600 complete the study.

### Operational definition and measurements

#### Cultural malpractice

 Any cultural practice that can lead to an injurious, negligent, or improper practice that is accepted by a certain community [31]. Cultural malpractice was considered as yes if the woman responded “Yes” when asked whether she practiced each malpractice (i.e. home delivery, abdominal massage, newborn bath soon after birth, pre-lacteal feeding, and colostrum avoidance) during childbirth otherwise considered as no [1, 21, 22, 24, 33, 34]. The overall magnitude was considered if the woman answered ‘‘Yes’’ at least one of the aforementioned cultural malpractices.

#### Antenatal care

 The care provided to all pregnant women by qualified health professionals during pregnancy regardless of prerequisites [35].

#### Cultural practices

 Experiences or beliefs that are socially shared views and behaviors practiced in a certain society at a certain time [24].

Home delivery is defined when women delivered at home or gave birth in a residence rather than a birthing center by unskilled birth attendants with culturally acceptable ceremonies [36, 37].

**Colostrum**; is yellowish and sticky breast milk produced in late pregnancy and the first few days after delivery and discarding of colostrum during the first five days after childbirth is considered as colostrum avoidance [34, 38].

**Pre-lacteal feeding**; giving of fluid or semisolid food before breastfeeding to an infant during the first three days after birth [39, 40].

#### Abdominal massage

 Hand-based downward rubbing of the pregnant woman’s abdomen to shorten the labor [21].

#### Newborn bathing

The immersion of all or part of the body of a newborn in water or some other liquids for cleansing or refreshment before 24 h after delivery [38].

### Data collection tool, procedures, and quality assurance

Data were collected using a semi-structured, pre-tested, and interview-based questionnaire. It was prepared in English and translated into the local language (i.e. Amharic) and finally returned to English. Pretest was done on 5% of the sample size and the necessary revision was then made on the tools after the pretest result for further clarity. Six preparatory and three college students were involved in the data collection process after offering one day of training for data collectors and supervisors. During data collection, each data collector was supervised for any respective feedbacks.

### Data processing and analysis

All collected data were rechecked for completeness and coded. Then, data were entered using Epi Info seven software and exported to SPSS version 25 for analysis. For further analysis, descriptive statistics like frequencies and cross-tabulation were performed. Bivariable logistic regression was employed to identify the association, and a multivariable logistic regression model was used to control the effect of confounders. Hosmer-Lemeshow statistic was done for model fitness. Variables having P-value less than 0.2 in the bivariable analysis were fitted into the multivariable logistic regression model. Multicolinearity reduces the power of coefficients and weakens the statistical measure to trust the p-values to identify the independently significant variables. Hence, the multicolinearity problem was checked with the help of tolerance and variance inflation factor (VIF). Unfortunately, no variable was detected that has collinearity effect or variable which has tolerance test <0.1 and VIF>10. The 95% CI and odds ratio were computed and variables having a p-value less than 0.05 in the multivariable logistic regression analysis were considered to declare statistical significance association.

## Results

### Socio-demographic characteristics

Of the sampled 617 participants, 600 took part in the study making the response rate 97.25%. The mean age of the study participants was 30.35 years (SD± 5.08 years) ranging from 18 to 47 years. Most of the participants (95.2%) were Orthodox Christianity followers. Four hundred ninety-six (82.7%) of the study participants belong to the age group between 20 and 35 years. Likewise, regarding the occupation of mothers and marital status, 87.8% and 96.8% were housewives and married respectively. Moreover, regarding educational status, 55.8% of women unable to read and write and only 6.0% of them were accomplished college and above level of education [See Table 1].

**Table 1:** Socio-demographic characteristics of respondents among women who give birth within one year in rural Gozamen district, Amhara region, Northwest Ethiopia,2019 (*n* = 600)

Variables	Frequency	Percent
**Maternal age**		
≤19 year	4	0.7
20-35 year	496	82.7
≥36 year	100	16.6
**Religion**		
Orthodox	571	95.1
Muslim	25	4.2
Protestant	4	0.7
**Ethnicity**		
Oromo	6	1.0
Amhara	587	97.8
Other	7	1.2
**Mother’s occupation**		
housewife	527	87.8
Gov’t employer	28	4.7
Merchant	42	7.0
Student	3	0.5
**Marital Status**		
Married	581	96.8
Single	4	0.7
Divorced	15	2.5
**Mother’s educational status**		
Unable to read and write	335	55.8
Primary school	149	24.8
Secondary school	80	13.4
College and above	36	6.0
**Husband’s occupation**		
Farmer	413	71.1
Government employer	51	8.8
Merchant	83	14.3
Student	2	0.3
Daily laborer	32	5.5
**Husband’s educational status**		
Unable to read and write	328	56.5
Primary school	127	21.8
Secondary school	76	13.1
College and above	50	8.6

### Obstetrical characteristics of respondents

In this study, 87.5% of the respondents had ANC follow-up for their last pregnancy. Regarding party, 77% were multiparous and 49.5% of the respondents were given 2–4 childbirth. Just 67.1% of the participants gave birth at a health facility by a trained health professional during the last childbirth whereas 33.9% of the participants were delivered at home. Out of 33.9% of mothers who delivered their last child at home; 41.38%, 35.96%, and 22.66%, of them were assisted by their family members, traditional birth attendants (TBAs), (and neighbors respectively. Of home deliveries, 88.17% of the participants used a new blade and 6.4% used unclean blade to cut the umbilical cord [See Table 2].

**Table 2:** Obstetric related characteristics of respondents among women who gave birth within the last one year in rural Gozamen district, Northwest Ethiopia, 2019 (*n* = 600)

Obstetrics variables	Frequency	Percent
**Parity**		
Parity 1	138	23.0
Parity 2-4	297	49.5
Parity=>5	165	27.5
**ANC Visit**		
Yes	525	87.5
No	75	12.5
Number of ANC follow up (n = 525)		
One	98	18.67
Two	136	25.9
Three and above	291	55.43
**Place of delivery**		
Home	203	33.9
Health facility	397	66.1
**If at home, delivery assistance was (n = 203)**		
Traditional birth attendants	73	35.96
Family member	84	41.38
Neighbor	46	22.66
**An instrument used to cut the cord (n = 203)**		
New blade	179	88.17
Unclean blade used before	13	6.4
Boiled blade used before	11	5.43
**Umbilical cord tied**		
Yes	156	76.85
No	47	23.15

Key: *ANC *Antenatal care

### Cultural malpractices during childbirth and postpartum period

Generally, in the Gozamen district, 31.2% of mothers are practiced cultural malpractice during their childbirth. Of these, 33.9% of the participants delivered at their home, 11.2% applied abdominal massage with butter to facilitate labor. Moreover, cultural malpractices like abdominal massage 11.2%, home delivery 33.9%, Regarding breastfeeding, 12.8% of the participants reported that they were provided pre-lacteal feeding and 16.6% of the participants discarded the colostrum. Likewise, 23.0% of the participants have washed their babies soon after birth and or within 24 h of birth [See Table 3].

**Table 3:** Prevalence of cultural malpractices during childbirth and postpartum among women who gave birth within the last one year in rural Gozamen district, Northwest Ethiopia, 2019 (*n* = 600)

Cultural malpractice	Frequency	Percent
Abdominal massage	67	11.2
Home delivery	203	33.9
Colostrum avoidance	31	16.6
Pre-lacteal feeding	24	12.8
Bath newborn soon after birth	138	23
Overall at least one cultural malpractice	187	31.2

### Factors associated with the response variable

Bivariate analysis showed that: mothers’ occupation, married marital status, unable to read and write participant educational status and husband (unable to read and write and primary level of education), farmer husband occupation, parity ≥5, and having ANC visit was crudely associated. Independently associated variables in adjusted analysis were: Married marital status, being farmer husband occupation, parity ≥5, and having ANC visit.

Accordingly, this study found that those women with married marital relations were 76% less likely to exercise cultural malpractices during childbirth (AOR= 0.24; 95% CI: 0.10, 0.89) and being farmer husband occupation was 5.1 (AOR= 5.1; 95% CI: 1.15, 13.02) times more likely to use cultural malpractices as compared to those divorced and daily laborer respectively. This study also established that the parity of women ≥5 was 4.87 times more likely to use cultural malpractices during their childbirth as compared to primipara women (AOR= 4.87; 95% CI: 2.44, 9.52).

Further, ANC visit is an important restraining significant factor for cultural malpractices. Thus, those women who have ANC follow-up delivery were 48% less likely to use cultural malpractices for their childbirth as compared to their counterparts (AOR= 0.52; 95% CI: 0.28, 0.94) (See Table 4).

**Table 4:** Bivariable and multivariable logistic regression analysis of factors associated with cultural malpractice during childbirth and postpartum period among women who give birth within the last one year in rural Gozamen district, Northwest Ethiopia 2019 (*n* = 600)

Variables	Cultural malpractice Yes No		COR (95% CI)	AOR(95% CI)
**Mothers occupation**				
House wife	180	347	0.25(0.06,27.51)	0.06(0.05, 22.54)
Government employer	2	26	0.03(0.02,0.63)	0.08(0.06, 17.75)
Merchant	3	39	0.03(0.02,0.57)	0.10(0.08, 15.98)
Student	2	1	1	1
**Marital status**				
Married	174	407	0.21 (0.07,0.63)*	0.24 (0.10, 0.89)*
Single	3	1	1.50 (0.36,54.86)	9.09 (0.11,50)
Divorced	10	5	1	1
**Mothers education**				
Unable to read and write	152	183	9.13 (2.77, 33.33)	0.76 (0.10, 5.88)
Primary school	25	124	2.21 (0.63, 8.33)	0.33 (0.04, 2.63)
Secondary	7	73	1.05 (0.69, 11.74)	0.40 (0.05, 3.57)
College and above	3	33	1	1
**Husband occupation**				
Farmer	167	246	10.18 (2.4, 43.18)*	5.1 (1.15, 13.02)*
Government employer	1	51	0.30 (0.03, 3.57)	1.25 (0.02, 16.67)
Merchant	5	78	0.96 (0.48, 14.31)	0.56 (0.08, 4.0)
Student	1	1	15 (0.71, 23.33)	14.28 (0.33, 25)
Daily laborer	2	30	1	1
**Husband education**				
Unable to read and write	140	188	18.87 (5.26, 36)	11.11(0.24, 29)
Primary school	43	76	13.59 (2.22, 28)	12.5 (0.27, 34)
Secondary	17	59	6.92 (0.69,50)	9.11 (0.24, 47)
College and above	2	48	1	1
**Parity**				
Para 1	18	120	1	1
Para 2-4	65	232	1.86(1.06,3.33)	1.31(0.69, 2.48)
Para ≥5	104	61	11.36 (6.67,25)**	4.87 (2.44, 9.52)**
**ANC Visit**				
Yes	155	369	0.58 (0.35, 0.95)*	0.52 (0.28, 0.94)*
No	32	44	1	

Key: **=*p*-value<0.001, *=*p*-value<0.05, *COR *Crude odds ratio; *AOR *Adjusted odds ratio; 1 = Reference category

## Discussion

This community-based study has attempted to assess the prevalence of cultural malpractice and associated factors during childbirth and postpartum among women who gave birth within one year, in Gozamen district, Ethiopia. The overall magnitude of cultural malpractices was found to be 31.2%. Of participants, 33.9% delivered at home, 11.2% experienced abdominal massage, 16.6% avoided colostrum, 12.8% practiced pre-lacteal feeding and 23.0% washed their baby before 24 h after delivery. Moreover, significant factors associated with cultural malpractice during childbirth and postnatal period were; mothers who have antenatal follow-up, married marital relation, being farmer husband occupation, and parity ≥5.

The overall finding of this study (31.2%; 95% CI: 27.8, 34.7) was lower than the studies done at Meshenti town (37.9%) [1], Axum (87.8%) [40]. The difference can be explained by the discrepancy in the background of the study participants, study settings, time gap, and the availability and accessibility of the infrastructures. In addition to this, the health system-related factors might be contributing to this variation due to the extensive work of health extension workers and various health care institutions in awareness creation about the drawback of harmful cultural practices in the study area.

The result of this study revealed that the prevalence of home delivery was 33.9%. The finding is in line with a study conducted in Northwest Ethiopia (32.3%) [5]. This islower than the study conducted in Arba Minch (79.4%) [29], Zala woreda (67.6%) [41], 2019 Mini-EDHS report which is 52% [42], and Limmu Genet Town (38.3%) [22]. The probable reason for the difference may be socioeconomic and cultural factors that may vary among the studies. Cultural and traditional factors play a major role that enhances giving birth at home and TBAs are preferred by women for their birth assistance at home [30, 43]. However, this finding is higher as studies conducted from Meshenti Town (29.7%) [1], Serra Leon (31.1%) [42], and Kenya (26%) [44]. Out of 33.9% of mothers who delivered their last child at home, most of them were assisted by their family members at 41.38%, TBAs at 35.96%, and neighbors at 22.66%. Of home delivered participants, 88.17% was used new blade and 6.4% used unclean blade to cut the umbilical cord and also about 23.15% of the mother who delivered at home umbilical cord was not tied.

Our study revealed that the prevalence of abdominal massage was 11.2% which was lower than that of the study conducted in Lemmu Genet Town at 22% [22] and Niger at 70.5% [21]. However, this finding is higher than the studies conducted in Meshenti Town and Northwest Ethiopia, where the prevalence was (2.73%) [1] and 9.1% [5] respectively. The possible reason for this difference could be the source of the sampled population and cultural diversity throughout the country.

Colostrum is still discarded in different parts of the globe, particularly in Ethiopia. This investigation revealed that the prevalence of avoiding colostrum was 16.6% which was higher than the study conducted in Raya Kobo district at 13.5% [28], Southern Ethiopia at 8.5% [45], and Axum Town at 6.3% [43]. But the current finding was lower than the study conducted in Limmu Genet at 22.41% [22], in rural Northern Ethiopia at 79% [46], and 22.1% in Northwest Ethiopia [43]. This difference might be due to socio-cultural and infant feeding styles that affect colostrum feeding since colostrum avoidance is traditional malpractice affected by the attitude and cultural beliefs of the community. Similarly, the prevalence of pre-lacteal feeding practice was 12.8%. This might be due to misperception of the mothers and sociocultural malpractices of early breastfeeding. Higher findings reported from Ethiopia (20.6-63%) [34, 38, 47], India (88%) [36] and Nigeria (26.3%) [37]; however, slightly similar with a study conducted Burie district 11.6% [48]. Thus, the practice of giving pre-lacteal feed to babies is greatly affected by a traditional culture [36]. The possible explanation for the observed discrepancy could be due to differences among study settings.

The leading thermal practice by women of developing countries is early bathing which predisposes newborns for life-threatening situations like hypothermia, low blood sugar levels, respiratory distress, abnormal clotting, jaundice, pulmonary hemorrhage) and increased risk of developing infections. This finding also provided evidence regarding newborns were given a bath soon after birth before 24 h after delivery (23%) that is lower than the research done at Lemmu Genet Town (28.4%) [22], Meshenti Town (45.2%) [1], Harari Region 35% [38] and Nepal (31%) [24]. This may have been explained as the socio-cultural difference among study participants and time gap as efforts by the Ethiopian government of increasing health education and communication outreach programs access to mothers especially in rural areas.

Literature from various sources has enunciated on the importance of maternal health care services like ANC; importantly this study highlights on the importance of ANC follow-up visits. Participants who had ANC follow-up were 48% less likely to use cultural malpractice than those who had no ANC follow-up. This could be explained by medical examination during pregnancy can help women with information about the merits of delivery in the presence of a skilled birth attendant, to be guided recognizing symptoms of complications early enough and act accordingly to ward off any potential danger promptly [49]. This finding is in agreement with the study done in other parts of Ethiopia [5, 45], Tanzania [50], and Zambia [51]. Thus, care before birth is a necessary intervention for achieving healthy outcomes for pregnancy by providing counseling and health information about the complication of cultural malpractice [52].

The odds of having cultural malpractice in mothers who were married were 76% times less likely to use cultural malpractice during childbirth and postnatal period than those who were divorced which is highly consistent with existing literature [53–57]. This could be explained by in divorced women, obtaining necessities such as food and shelter, and relationships and social, emotional support might be compromised. Hence, mothers who were married were supported by their husbands to made decisions and went to health facilities which tend to discourage cultural malpractice [57]. Moreover, many pregnancies among single women being unplanned, and for unplanned/unintended pregnancies, ANC follow-up is improbable and family pressures may keep them at home because pregnancy before marriage is shameful for the family. On the other hand, this finding is inconsistent with a study in Tigray regional state [58] which showed unmarried women who were likely to deliver in a facility tended to have seldom cultural malpractice during perinatal period as compared with married ones.

Importantly, husbands´ occupation was associated with cultural malpractice during childbirth and postpartum. Women who classified their husbands´ occupation as “farmer’’ were more likely to use cultural malpractices than daily laborer’ wives. We could not find any previous studies comparing factors associated with cultural malpractice disaggregated by farmer husband occupation in Ethiopia. However, studies demonstrated significant associations of cultural malpractice during childbirth and postpartum with the husband’s educational status [5, 59]. This could be justified as farmers often might have no awareness about the disadvantage of harmful malpractice during perinatal period and they might spend their time far from the healthcare facility in farming areas, may face more difficulty of transportation. This finding was comparable to the results of another study where women with husbands in non-farming occupations were more likely to use maternal healthcare services [60]. 

In the present study, grand multiparity appears as factor responsible for the utilization of cultural malpractice than primipara. This result was consistent with other national and elsewhere studies [5, 59–61,62]. The reason could be a better understanding of the advantages of maternal healthcare in young mothers. In addition, the lower parity mothers might have less experience may develop fear about the difficulties during labor whereas multiparous women who had done their previous deliveries at home assisted by TBAs and/or unskilled providers were more likely to admit cultural malpractice [60, 61, 63]. Aspects of traditional beliefs, personal experiences of mothers, and the perspectives of community members were among possible reasons cited for this outcome [24, 30].

However, this study addressed several cultural malpractices during childbirth and postpartum with enough sample size to generalize the findings, the authors acknowledged the following limitations; first, the cross-sectional nature does not allow establishing causality of associations and the results should be interpreted cautiously. Second, recall bias cannot be ruled out about events that took place for the last 12 months’ exposure. Third, however, socially desirable responses were minimized with separate and private interview areas it may also be a problem. Lastly, the final fate of cultural malpractice during childbirth on the mother as well as on the child was not assessed.

## Conclusions

Cultural malpractices are common in rural population in Ethiopia. Further qualitative studies are needed to explore the behavioral reasons for traditional malpractices during childbirth and postpartum. Community-based interventions are required to improve the number of families engaging ANC and a skilled birth attendant during pregnancy and delivery respectively. The high-risk traditional childbirth and newborn care practices like home delivery, abdominal massage, discarding colostrum, pre-lacteal feeding and washing baby soon after birth before 24 h need to be addressed by culturally acceptable community-based health education programs.

## Data Availability

The datasets used and/or analyzed during the current study are available from the corresponding author on reasonable request.

## Abbreviations

ANC: Antenatal care
EDHS: Ethiopian Demographic Health Survey
HTPs: Harmful Traditional Practices
TBA: Traditional Birth Attendants

## References

[1] Tsegaw A, Debebe E, “The Prevalence of Traditional Malpractice during Pregnancy, Child Birth, and Postnatal Period among Women of Childbearing Age in Meshenti Town, 2016,” Int. J. Reprod. Med., vol. 2018:7, 2018, [Online]. Available: 10.1155/2018/5945060. 10.1155/2018/5945060 PMC5820650 29568739

[2] Parties S, et al., “Fact Sheet No. 23, Harmful Traditional Practices Affecting the Health of Women and,” no. 23, 1995.

[3] Behruzi R, Hatem M, Fraser W, Goulet L, Ii M, Misago C. “Facilitators and barriers in the humanization of childbirth practice in Japan,” BMC Pregnancy Childbirth. 2010;10;25:18, [Online]. Available: http://www.biomedcentral.com/1471-2393/10/25. 10.1186/1471-2393-10-25 PMC2889847 20507588

[4] Morris JL, Short S, Robson L, Andriatsihosena MS (2014). Maternal Health Practices, Beliefs and Traditions in Southeast Madagascar. Afr J Reprod Health.

[5] Melesse MF, Bitewa YB, Dessie KN, Wondim DB, Bereka TM (2021). Cultural malpractices during labor/delivery and associated factors among women who had at least one history of delivery in selected Zones of Amhara region, North West Ethiopia: community based cross-sectional study. BMC Pregnancy Childbirth.

[6] Gebrekirstos K, Fantahun A, Buruh G, “Magnitude and Reasons for Harmful Traditional Practices among Children Less Than 5 Years of Age in Axum Town, North Ethiopia, 2013,” Int. J. Pediatr*. *2014;2014:1–5, 2014, doi: 10.1155/2014/169795. 10.1155/2014/169795 PMC4089850 25045359

[7] Abubakar S, Adamu D, Hamza R, Galadima JB (2017). Review of Quality of Care Determinants of Home Delivery among Women attending Antenatal Care in Bagwai Town, Kano Nigeria. Afr J Reprod Health.

[8] Ajiboye OE, Abimbola AK (2012). Socio-cultural factors affecting pregnancy outcome among the Ogu speaking people of Badagry area of Lagos State, Nigeria. Int J Humanit Soc Sci.

[9] World Health Organization (WHO). WHO reccommendations on antenatal care for a positive pregnancy experience. 2016. 28079998

[10] Tekelab T, Chojenta C, Smith R, Loxton D. “Factors affecting utilization of antenatal care in Ethiopia: A systematic review and meta- analysis,” PLoS One, pp. 1–24, 2019. 10.1371/journal.pone.0214848 PMC6459485 30973889

[11] Henok A (2015). “Knowledge, Attitude and Practice of Antenatal Care Service among Married Women of Reproductive Age Group in Mizan Health Center, South West Knowledge. J Med Physiol Biophys.

[12] Afaya A (2020). Women ’ s knowledge and its associated factors regarding optimum utilisation of antenatal care in rural Ghana: A cross- sectional study. PLoS ONE.

[13] Abdulrida H, Hassan R, Sabri M (2018). Knowledge and health-seeking practices of mothers attending primary health-care centers in baghdad al-karkh sector about danger signs in newborns. Mustansiriya Med J.

[14] Tilahun T, Sinaga M, “Knowledge of obstetric danger signs and birth preparedness practices among pregnant women in rural communities of Eastern Ethiopia,” Int. J. Nurs. Midwifery. 2016;8:1–11. doi: 10.5897/IJNM2015.0199.

[15] Ó¦zge Tunçalp and A. P. and AMG Juan Pablo Pena-Rosas, T Lawrie, Bucagu M, Olufemi T.Oladapo, “WHO recommendations on antenatal care for a positive pregnancy experience — going beyond survival,” Int. J. Obstet. Gynecol. 2017: doi: 10.1111/1471-0528.14599. 10.1111/1471-0528.14599 28190290

[16] WHO U. UNICEF, Trends in maternal mortality 2000 to 2017: estimates by WHO, UNICEF, UNFPA, World Bank Group and the United Nations Population Division. Geneva: World Health Organization; 2019: Licence: CC BY-NC-SA 3.0 IGO., 2017.

[17] World Health Organization (WHO). World Health Statitics, vol. 13, no. 3. 2015.

[18] UNICEF. UNICEF annual report 2018. 2018.

[19] Zhao Q, Kulane A, Gao Y, Xu B (2010). Knowledge and attitude on maternal health care among rural-to-urban migrant women in Shanghai, China. BMC Women ’ s Heal.

[20] Id HA, Beyene GA, Mulat BS (2021). Harmful cultural practices during perinatal period and associated factors among women of childbearing age in Southern Ethiopia: Community based cross-sectional study. PLoS ONE.

[21] Addah IE, Ibrahim AO (2021). The prevalence and predictors of abdominal massage among pregnant women attending antenatal care at the Niger Delta university teaching hospital, Okolobiri, Nigeria. Niger Heal J.

[22] Tadesse NT, Tadesse AH (2015). Cultural Malpractices During Pregnancy, Child Birth and Postnatal Period Among Women of Child Bearing Age in Limmu Genet Town, Southwest Ethiopia. Sci J Public Heal.

[23] Liamputtong P, Yimyam S, “Traditional beliefs about pregnancy and child birth among women from Chiang Mai, Northern Thailand,” *Midwifery*, vol. 21, no. June 2014, pp. 139–153, 2009, doi: 10.1007/978-90-481-2599-9. 10.1016/j.midw.2004.05.002 15878429

[24] Chand SB (2016). Cultural beliefs and traditional rituals about child birth practice in rural, Nepal. MOJ Public Heal.

[25] UNAM OHCHRE. Harmful Traditional Practices and Implementation of the Law on Elimination of Violence against Women in Afghanistan, no. December. 2010.

[26] Kuruvilla S, et al., “(2016 – 2030): a roadmap based on evidence and country experience,” World Heal. Organ., vol. 34, no. March, pp. 398–400, 2016, [Online]. Available: doi: http://dx.doi.org/10.2471. 10.2471/BLT.16.170431 PMC4850541 27147772

[27] Maezu G, Azene ZN, Mulunesh A, Alamneh TS (2021). Delayed breast feeding initiation increases the odds of colostrum avoidance among mothers in Northwest Ethiopia: a community-based cross-sectional study. Arch public Heal.

[28] Legesse M, Demena M, Mesfin F, Haile D, “Factors Associated with Colostrum Avoidance Among Mothers of Children Aged less than 24 Months in Raya Kobo district, North-eastern Ethiopia : Community-based Cross-sectional Study,” no. July, pp. 357–363, 2015, doi: 10.1093/tropej/fmv039. 10.1093/tropej/fmv039 PMC4590259 26141533

[29] Ayele G, Tilahune M, Merdikyos B, Animaw W, Taye W (2015). Prevalence and associated factors of home delivery in Arbaminch Zuria district, southern Ethiopia : Community based cross sectional study. Sci J Public Heal.

[30] Mwewa CMD (2010). Factors associated with home deliveries in a low income rural setting-observations from Nchelenge district, Zambia. Med J Zambia.

[31] Sharma S, Van Teijlingen E, Hundley V, Angell C, Simkhada P (2016). Dirty and 40 days in the wilderness: Eliciting childbirth and postnatal cultural practices and beliefs in Nepal. BMC Pregnancy Childbirth.

[32] Abegunde D (2017). Trends in newborn umbilical cord care practices in Sokoto and Bauchi States of Nigeria: the where, who, how, what and the ubiquitous role of traditional birth attendants : a lot quality assurance sampling survey. BMC Pregnancy Childbirth.

[33] Mugyenyi GR, Obua C, Agaba E, Ware NC, Matthews LT (2020). When Women Deliver at Home Without a Skilled Birth Attendant: A Qualitative Study on the Role of Health Care Systems in the Increasing Home Births Among Rural Women in Southwestern Uganda. Int J ofWomen’s Heal.

[34] Devkota B (2016). Determinants of home delivery in Nepal – A disaggregated analysis of marginalised and non-marginalised women from the 2016 Nepal Demographic and Health Survey. PLoS ONE.

[35] WHO. “WHO recommendations on maternal health guidelines approved by the WHO guidelines review committee.Geneva: World Health Organization; 2017 (WHO/MCA/17.10). Licence: CC BY-NC-SA 3.0 IGO.,” no. May, 2017. 40455892

[36] Raina S, Mengi V, Singh G (2012). Determinants of prelacteal feeding among infants of RS Pura block of Jammu and Kashmir, India. J Fam Med Prim Care.

[37] Ogundele T, Ogundele OA, Adegoke AI (2019). Determinants of prelacteal feeding practices among mothers of children aged less than 24 months in ile-ife Southwest Nigeria: A community cross-sectional study. Pan Afr Med J.

[38] Welay FT (2020). Early Newborn Bath and Associated Factors among Parturient Women Who Gave Birth in the Last Month in Harar Region, Eastern Ethiopia, 2017. ” Open Public Health J.

[39] E. Central Statistical Agency Addis Ababa, T. D. P. ICF, and U. Rockville, Maryland, *Ethiopia Demographic and Health Survey key indicators*. 2016.

[40] G. K., A. M., and F. A., “A cross sectional study on factors associated with harmful traditional practices among children less than 5 years in Axum town, north Ethiopia, 2013.Reprod. Health, vol. 11, no. 1, pp. 1–7, 2014, [Online]. Available: http://www.embase.com/search/results?subaction=viewrecord&from=export&id=L53202178%0Ahttp://dx.doi.org/10.1186/1742-4755-11-46. 10.1186/1742-4755-11-46 PMC4082281 24952584

[41] Kucho B, Mekonnen N (2017). Delivery at home and associated factors among women in child bearing age, who gave birth in the preceding two years in Zala Woreda, southern Ethiopia. J. Public Heal. Epidemiol..

[42] Sharkey A, et al., “Maternal and newborn care practices in Sierra Leone: a mixed methods study of four underserved districts,” Health Policy Plan., vol. 32, no. August 2016, pp. 151–162, 2017, doi: 10.1093/heapol/czw104. 10.1093/heapol/czw104 28207047

[43] Weldesamuel GT, Atalay HT, Zemichael TM, Gebre HG (2018). Colostrum avoidance and associated factors among mothers having children less than 2 years of age in Aksum town, Tigray, Ethiopia : a cross – sectional study 2017. BMC Res Notes.

[44] Moindi RO, Ngari MM, Nyambati VCS, Mbakaya C (2016). Why mothers still deliver at home: understanding factors associated with home deliveries and cultural practices in rural coastal Kenya, a cross-section study. BMC Public Health.

[45] Amele EA, Demissie BW, Desta KW, Woldemariam EB (2019). Prelacteal feeding practice and its associated factors among mothers of children age less than 24 months old in Southern Ethiopia. Ital J Pediatr.

[46] Rogers NL (2011). Colostrum avoidance, prelacteal feeding and late breast-feeding initiation in rural Northern Ethiopia. Public Health Nutr.

[47] Bililign Yimer N, Tenaw Z, Solomon K, Mulatu T, “Inadequate prenatal visit and home delivery as determinants of perinatal outcomes: does parity matter?,” *J. Pregnancy*, vol. 2019, 2019. 10.1155/2019/9024258 PMC6481024 31093374

[48] Gebremeskel SG, Gebru TT, Kassahun SS, Gebrehiwot BG, “Magnitude of Prelacteal Feeding and Its Associated Factors among Mothers Having Children Less than One Year of Age: A Community-Based Cross-Sectional Study in Rural Eastern Zone, Tigray, Ethiopia,” Adv. Public Heal. 2020;2020:8–10.

[49] Choulagai B (2013). Barriers to using skilled birth attendants’ services in mid- and far-western Nepal: A cross-sectional study. BMC Int Health Hum Rights.

[50] Mohan D (2017). Analysis of dropout across the continuum of maternal health care in Tanzania: findings from a cross-sectional household survey. Health Policy Plan..

[51] Scott NA (2018). Factors affecting home delivery among women living in remote areas of rural zambia: A cross-sectional, mixed-methods analysis. Int J Womens Health.

[52] Kaso M, Addisse M (2014). Birth preparedness and complication readiness in Robe Woreda, Arsi Zone, Oromia Region, Central Ethiopia: A cross-sectional study. Reprod Health.

[53] He S, Chen S, Kong L, Liu W (2021). Analysis of Risk Perceptions and Related Factors Concerning COVID-19 Epidemic in Chongqing, China. J Community Health.

[54] Porch TC (2016). The role of marital status in physical activity among African American and White men. Am J Mens Health.

[55] Bayu H, Fisseha G, Mulat A, Yitayih G, Wolday M (2015). Missed opportunities for institutional delivery and associated factors among urban resident pregnant women in South Tigray Zone, Ethiopia: A community-based follow-up study. Glob Health Action.

[56] Gebregziabher NK, Zeray AY, Abtew YT, Kinfe TD, Abrha DT (2019). Factors determining choice of place of delivery: Analytical cross-sectional study of mothers in Akordet town, Eritrea. BMC Public Health.

[57] Pecoraro A (2013). Factors contributing to dropping out from and returning to HIV treatment in an inner city primary care HIV clinic in the United States. AIDS Care - Psychol Socio-Medical Asp AIDS/HIV.

[58] T. Y., G. T., G. I., E. K., L. H., and S. M.S., “Determinants of antenatal and delivery care utilization in Tigray region, Ethiopia: A cross-sectional study,” *Int. J. Equity Health*, vol. 12, no. 1, pp. 1–10, 2013, [Online]. Available: http://www.embase.com/search/results?subaction=viewrecord&from=export&id=L52585240%0Ahttp://dx.doi.org/10.1186/1475-9276-12-30. 10.1186/1475-9276-12-30 PMC3658893 23672203

[59] Gabrysch S, Campbell OMR (2009). Still too far to walk: Literature review of the determinants of delivery service use. BMC Pregnancy Childbirth.

[60] Dhakal P, Shrestha M, Baral D, Pathak S (2018). Factors affecting the place of delivery among mothers residing in Jhorahat VDC, Morang, Nepal. Int J Community Based Nurs Midwifery.

[61] Ogunyomi MT, Ndikom CM (2016). Perceived Factors Influencing the Utilization of Traditional Birth Attendants ’ Services in Akinyele Local Government, Ibadan, Nigeria. J Community Med Prim Heal Care.

[62] Omaka-amari LN, Obande-ogbuinya EN, Aleke CO, Eunice AN, Nwafor JN, Nwankwo O (2021). Demographic Predictors of Cultural Practices Regarding Female Genital Mutilation among Married Women in Ebonyi State, Nigeria. J Adv Med Med Res.

[63] Mazalale J (2015). Factors associated with delivery outside a health facility: Cross-sectional study in rural Malawi. Trop Med Int Heal.

